# Titania Photonic Crystals with Precise Photonic Band Gap Position via Anodizing with Voltage versus Optical Path Length Modulation

**DOI:** 10.3390/nano9040651

**Published:** 2019-04-23

**Authors:** Georgy A. Ermolaev, Sergey E. Kushnir, Nina A. Sapoletova, Kirill S. Napolskii

**Affiliations:** 1 Center for Photonics and Quantum Materials, Skolkovo Institute of Science and Technology, 143026 Moscow, Russia; georgy.ermolaev@skoltech.ru; 2 Center for Photonics and 2D Materials, Moscow Institute of Physics and Technology, 141700 Dolgoprudny, Russia; georgiy.ermolayev@phystech.edu; 3 Department of Chemistry, Lomonosov Moscow State University, 119991 Moscow, Russia; nina@inorg.chem.msu.ru (N.A.S.); kirill@inorg.chem.msu.ru (K.S.N.); 4 Department of Materials Science, Lomonosov Moscow State University, 119991 Moscow, Russia

**Keywords:** anodic titanium oxide, anodizing, effective refractive index, photonic crystals, photonic band gap, porous film

## Abstract

Photonic crystals based on titanium oxide are promising for optoelectronic applications, for example as components of solar cells and photodetectors. These materials attract great research attention because of the high refractive index of TiO_2_. One of the promising routes to prepare photonic crystals based on titanium oxide is titanium anodizing at periodically changing voltage or current. However, precise control of the photonic band gap position in anodic titania films is a challenge. To solve this problem, systematic data on the effective refractive index of the porous anodic titanium oxide are required. In this research, we determine quantitatively the dependence of the effective refractive index of porous anodic titanium oxide on the anodizing regime and develop a model which allows one to predict and, therefore, control photonic band gap position in the visible spectrum range with an accuracy better than 98.5%. The prospects of anodic titania photonic crystals implementation as refractive index sensors are demonstrated.

## 1. Introduction

Photonic crystals (PCs) have been a research focus since their discovery [1] due to the unusual interaction of electromagnetic waves with spatially-ordered structures they exhibit. Photonic crystal is a structure with a periodic dielectric constant [2] which forms a photonic band gap (PBG)—a certain range of wavelengths where light cannot propagate through matter. This feature has found many applications in photodetection [3], sensing [4], solar energy conversion [5], and waveguides [6]. Titanium oxide is a perspective material for creating PCs due to its transparency in a visible spectrum range and high refractive index (*n*_TiO2_ = 2.6 at *λ* = 600 nm [7]), which allows one to achieve a wide PBG.

Among the variety of approaches to the preparation of photonic crystals (e.g., self-organization of colloidal particles [8,9] and holographic lithography [10]), the most promising method for synthesis of titanium oxide photonic crystals is the anodizing of titanium under periodic anodizing conditions [11,12,13,14,15,16,17,18]. Such an approach allows one to prepare a one-dimensional PC by varying the anodizing voltage or current density leading to the change of porosity, *p*, and as a result, modulation of an effective refractive index [19], *n*_eff_, throughout the film thickness.

Fabrication of high-quality photonic crystals requires strict periodicity of *n*_eff_ as a function of the optical path length (OPL), which is the product of layer thickness, *d*, and *n*_eff_ of the medium through which light propagates. Earlier, a new anodizing regime with voltage versus electric charge density modulation *U*(*q*) was proposed for the fabrication of high-quality one-dimensional photonic crystals [14]. The key advantage of this regime over the voltage versus time *U*(*t*) [11,20] and current density versus time *j*(*t*) [17,21] anodizing regimes is highly precise control of the layers thickness due to the in situ measuring of charge. However, values of *n*_eff_ are required for the precise control of the OPL. The position of the photonic band gap, *λ*_0_, at the normal incidence of light is determined by

(1) $$m\lambda_{0}=2\left(d_{1}n_{1}+d_{2}n_{2}\right),$$

where *m* is the order of PBG, and *d*_1_ and *d*_2_ are the thicknesses of the successive layers inside the anodic titanium oxide (ATO) PC porous structure with the effective refractive indices *n*_1_ and *n*_2_, respectively [21]. To the best of our knowledge, there are no works demonstrating the possibility of prediction and precise control of photonic band gap position of ATO PCs.

Here, a new technique for the precise control of the thickness and *n*_eff_ of the layers of ATO PCs is developed. It has been shown that *n*_eff_ of anodic titanium oxide depends on the anodizing regime (constant or modulating voltage). Moreover, the suggested model takes into account the dissolution of ATO in electrolyte, usually resulting in the wider pore diameter at the top part of the oxide film in comparison with the bottom part [22,23,24].

## 2. Materials and Methods

### 2.1. Anodizing Conditions

The chemicals were purchased from Chimmed (Moscow, Russia) unless otherwise indicated. Before anodizing, titanium foils (99.6% purity, 0.15 mm thick, Auremo, Moscow, Russia) were electrochemically polished in the solution containing 15.6 M acetic acid and 1.0 M perchloric acid. Electropolishing was conducted at temperature below 25 °C for 4 min under square-wave applied voltage: 40 V for 10 s and 60 V for 10 s [14].

Ti was anodized in a two-electrode electrochemical cell (Figure S1 in Supplementary Materials). Electrolyte containing 0.08 M NH_4_F (Sigma-Aldrich, St. Louis, MO, USA) and 1.11 M H_2_O (obtained by water purification system MilliQ Integral 5, Millipore, Burlington, MA, USA) in ethylene glycol was chosen for anodizing because it allows one to prepare long and smooth titania nanotube arrays [25,26]. In electrolytes with higher concentration of NH_4_F ATO films with rough surface are formed [27]. The electrolyte was intensively stirred (480 rpm) using overhead stirrer and its temperature was kept constant at 30 °C. The distance between Ti cathode and anode was 1.1 cm in all experiments. The ATO films were prepared at constant voltages of 40 and 60 V and at modulating voltage. After synthesis, the ATO films were washed by ethanol and dried in air. It is worth noting that the as-prepared ATO porous films are amorphous [28,29]; thus, all characteristics of the samples given below demonstrate the properties of non-crystalline anodic titanium oxide.

### 2.2. Experiments at Constant Voltage

A number of samples were prepared at constant anodizing voltage of 40 and 60 V and the charge density of 2.33 C∙cmS^−2^. To investigate the chemical etching effect, the samples were exposed to the electrolyte under open circuit conditions for a different additional etching time (≤5 h) after the end of the anodizing.

The Fabry-Perot optical interference analysis of peak’s maxima [23,30] was used to determine the influence of the anodizing voltage (*U*), the etching time in the electrolyte solution (*t*_e_), and the charge density increment (Δ*q*) on the effective refractive index *n*_eff_(*U*, *t*_e_) and on thickness increment Δ*d*(Δ*q*, *U*) of ATO films. These dependencies were taken into account for the preparation of ATO PCs with precise PBG position by anodizing with voltage versus OPL modulation.

### 2.3. Fabrication of Photonic Crystals

Fabrication of PCs was carried out using voltage versus optical path length (*L*) modulation [31] by applying square-wave *U*(*L*) profile. The voltage was modulated between 40 and 60 V according to the following algorithm:

1. The OPL of one structure period, *L*_0_, was chosen with respect to the desired position of PBG at normal incidence of light on sample surface (*λ*_0_ = 2*L*_0_). Thus, the OPL of the whole ATO film is *L*_total_ = *N*·*L_0_*, where *N* is the number of structure periods.
2. The OPL of the layers obtained at 40 and 60 V was chosen equal to 0.5*L*_0_ unless otherwise indicated.
3. Total duration of synthesis, *t*_0_, was set before anodizing as a parameter. The value of *t*_0_ is the time between beginning of anodizing and the rinsing of the sample.
4. At the initial stage (*L* = 0 nm, *t* = 0 h), the anodizing voltage was set to 40 V.
5. After a short time-interval Δ*t* (ca. 30 ms), the charge density increment $\Delta q=\;\int_{t-\Delta t}^{t}j\left(t\right)dt$ was calculated, where *j*(*t*) is current density in the electrical circuit.
6. The increment of the OPL of ATO at wavelength *λ*_0_ is computed as a function of the charge density increment (Δ*q*), anodizing voltage (*U*), and the time of etching in the electrolyte (*t*_e_ = *t*_0_ – *t*): Δ*L* = OPL(Δ*q*, *U*, *t*_e_) = Δ*d*(Δ*q*, *U*)·*n*_eff_(*U*, *t*_e_).
7. A new value of the OPL is calculated as: *L*(*t*) = *L*(*t* – Δ*t*) + Δ*L*. Then, the voltage was set to the new value *U*(*L*(*t*)) which corresponds to the value of OPL in accordance with the required voltage—OPL profile.
8. Steps 5–7 were repeated until *L*(*t*) became higher than *L*_total_. After that, the power supply was switched off and the sample was exposed to the electrolyte under open circuit conditions until *t* > *t*_0_.

It should be noted that the OPL of the ATO film during anodizing means the OPL of the corresponding part of the ATO film with empty pores (filled by air) after the end of the synthesis.

### 2.4. Samples Characterization

The morphology of the ATO films was characterized by scanning electron microscopy (SEM) using an LEO Supra 50 VP instrument (Carl Zeiss SMT, Oberkochen, Germany). A Lambda 950 spectrophotometer (PerkinElmer, Waltham, MA, USA) was used for recording reflectance spectra in the range from 550 to 890 nm with the step of 1 nm at incident angles from 8° to 60°. The size of the light spot was 2 × 2 mm^2^. QE65000 spectrophotometer (Ocean Optics, Largo, FL, USA) was used for recording reflectance spectra of PCs at normal incidence of light in the range of 400–950 nm.

## 3. Results and Discussion

Optical interference fringes can be clearly seen on the reflectance spectra of the samples anodized at constant voltages (Figures S2 and S3 in Supplementary Materials). Thus, Fabry-Perot optical interference analysis [23,30] could be applied for determination of the dispersion of *n*_eff_ and thickness-to-charge density ratio, which are required for the developing of a quantitative model for the calculation of the OPL of ATO films.

The thickness-to-charge density ratio increases with anodizing voltage: the values of 876 ± 25 and 1054 ± 73 nm∙cm^2^∙C^−1^ were found for the samples anodized at 40 and 60 V, respectively. It is worth noting that the thickness-to-charge density ratios calculated from SEM images (Figure S4 in Supplementary Materials) are equal within the measurement accuracy to those obtained by the analysis of the reflectance spectra. The independence of the thickness-to-charge density ratio on time during anodizing of Al and Ti has been empirically proved earlier [14,23,32]. Consequently, assuming the linear increase in thickness-to-charge density ratio with anodizing voltage, the thickness increment of ATO films forming at constant voltage can be found as

(2) $$\Delta d^{c}\left(\Delta q,\;U\right)=\Delta q\cdot \left(d_{q}^{c}+d_{\mathrm{qU}}^{c}\cdot U\right),$$

where Δ*q* is the charge density spent for anodizing, *U* is the anodizing voltage, $d_{q}^{c}$ = 520 nm·cm^2^·C^−1^, $d_{\mathrm{qU}}^{c}$ = 8.9 nm·cm^2^·C^−1^·V^−1^. Here and below, the parameters for the case of constant voltage are marked by superscript index c.

**Figure 1:**
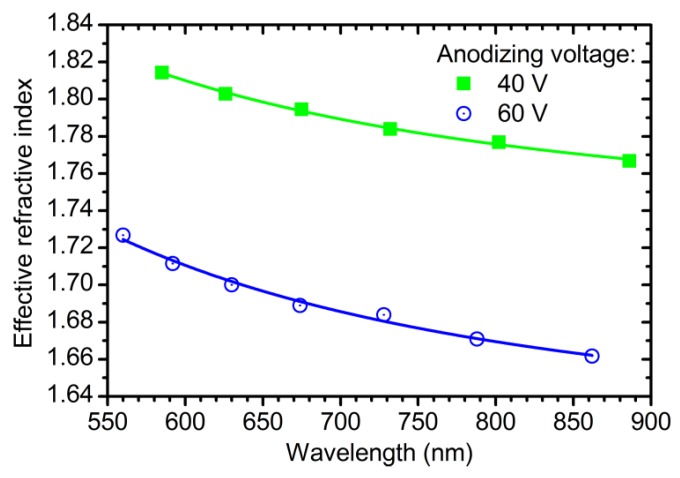
Wavelength dispersions of the effective refractive index of the anodic titanium oxide films obtained at 40 V (green squares) and 60 V (blue circles) without additional etching in electrolyte solution. Solid lines represent the fitting of experimental points by Cauchy dispersion.

Figure 1 shows the wavelength dispersion of *n*_eff_ for the samples obtained at 40 and 60 V without additional etching in electrolyte solution. The effective refractive index of ATO is lower than the refractive index of crystalline form of titania (*n*_TiO2_ = 2.6 at *λ* = 600 nm [7]) because *n*_eff_ decreases with porosity [19]. Experimental points are fitted well with the Cauchy dispersion:

(3) $$n^{c}\left(\lambda \right)=A^{c}+\;\frac{B^{c}}{\lambda^{2}},$$

This is usual for the region of transparency of semiconductors [33]. The fitting parameters are as follows: *A*^c^ = 1.732, *B*^c^ = 28,200 nm^2^ for the ATO film obtained at 40 V; *A*^c^ = 1.617; *B*^c^ = 33,840 nm^2^ for the sample prepared at 60 V. It should be noted that $n_{\mathrm{eff}}$ of ATO decreases with anodizing voltage, whereas $n_{\mathrm{eff}}$ of anodic aluminium oxide shows the reverse behaviour [22,31]. Such an opposite behaviour may be caused by weak dependence of the distance between centres of neighbouring pores in ATO on anodizing voltage (see Supplementary Materials “On ATO porosity” for additional information).

**Figure 2:**
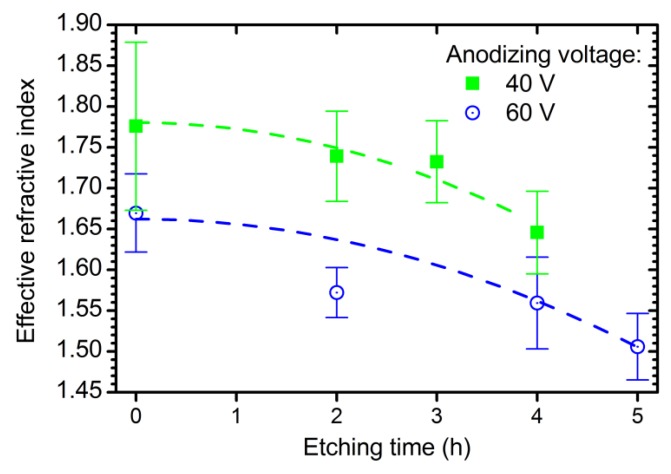
Dependences of the effective refractive index of the anodic titanium oxide at a wavelength of 800 nm on additional etching time in electrolyte solution. Experimental data for the samples obtained at 40 V (green squares) and 60 V (blue circles) are shown. Dashed lines represent the fitting of experimental data by empirical function (Equation (4)).

The effective refractive index of ATO decreases with the additional etching time (Figure 2) due to chemical pore widening and, as a consequence, growth of porosity [22,24]. The dependence of the effective refractive index of ATO films on time is fitted using the following equation (see dashed lines in Figure 2):

(4) $$n_{\mathrm{eff}}^{c}\left(U,t_{e}\right)=n_{0}^{c}-n_{U}^{c}\cdot U-n_{t}^{c}\cdot t_{e}^{2}+n_{\mathrm{Ut}}^{c}\cdot U\cdot t_{e}^{2},$$

where $n_{0}^{c}$ = 1.962; $n_{U}^{c}$ = 5.75 × 10^−3^ V^−1^; $n_{t}^{c}$ = 10.8 × 10^−3^ h^−2^; and $n_{\mathrm{Ut}}^{c}$ = 7.5 × 10^−5^ V^−1^h^−2^.

**Figure 3:**
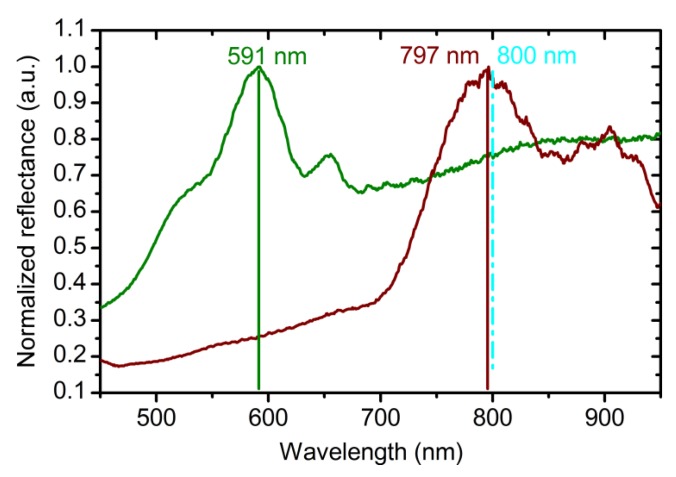
Reflectance spectra of anodic titanium oxide photonic crystals recorded at normal incidence of light. (Green line) PC_1 (PC—photonic crystal) was synthesized using Equations (2) and (4) for the optical path length (OPL) calculation. Parameters of the synthesis: *t*_0_ = 50 min; *L*_total_ = 20000 nm; *L*_0_ = 400 nm. (Red line) PC_2 was prepared using Equations (5) and (6) for the OPL calculation. Parameters of the synthesis: *t*_0_ = 50 min; *L*_total_ = 16000 nm; *L*_0_ = 400 nm. Vertical cyan dashed line and solid lines show the expected and experimental photonic band gap positions, respectively.

Dependences of $n_{\mathrm{eff}}^{c}\left(U,\;t_{e}\right)$ and $\Delta d^{c}\left(\Delta q,U\right)$ (Equations (2) and (4)) were used for the fabrication of the ATO photonic crystals via the algorithm described in the experimental section. The spectrum of the obtained PC (sample PC_1) is shown in Figure 3 (see green line). As can be seen, PBG is in shorter wavelength region than it has been expected. The average value of the ratio between the expected and experimentally observed PBG position is 1.34 (Figure 3). Thus, to achieve the consistency of the suggested model, for the layers fabricated at 40 and 60 V the values of OPL, calculated as a product of Equations (2) and (3), were divided by this factor to obtain more precise OPL^*^ values:

(5) $${\mathrm{OPL}}^{*}\left(\Delta q,\lambda ,U=40V\right)\;=\;\Delta q\cdot \left(A_{40}^{*}+\;\frac{B_{40}^{*}}{\lambda^{2}}\right),$$

(6) $${\mathrm{OPL}}^{*}\left(\Delta q,\lambda ,U=60V\right)\;=\;\Delta q\cdot \left(A_{60}^{*}+\;\frac{B_{60}^{*}}{\lambda^{2}}\right),$$

where $A_{40}^{*}$ = 1132 nm·cm^2^·C^−1^, $B_{40}^{*}$ = 1.844 × 10^7^ nm^3^·cm^2^·C^−1^, $A_{60}^{*}$ = 1272 nm·cm^2^·C^−1^, $B_{60}^{*}$ = 2.662 × 10^7^ nm^3^·cm^2^·C^−1^.

The reflectance spectrum of the photonic crystal PC_2 prepared using Equations (5) and (6) for the OPL calculation is shown in Figure 3. A photonic band gap near 800 nm is clearly seen. The difference between the expected and experimentally observed positions of the PBG is only 3 nm (<0.4%). It proves the correctness of Equations (5) and (6) for the calculation of *L*_0_ of the ATO photonic crystal for the case of equal assigned OPL of the layers formed at various voltages. 

**Figure 4:**
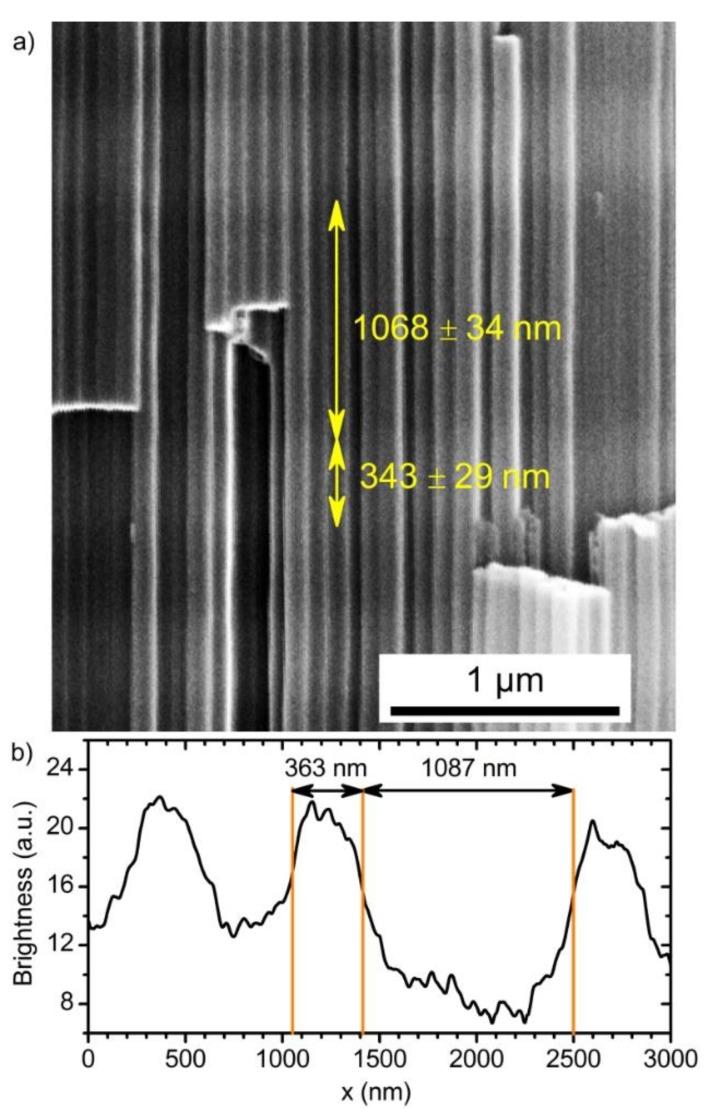
Morphology of porous anodic titanium oxide PC. (**a**) SEM image of a cleavage of the sample PC_3 with one thick layer obtained at 60 V. (**b**) An example of brightness profile of randomly selected nanotube from the panel in (**a**).

Close values of the measured and assigned position of the PBG of PC_2, obtained taking into consideration the OPL* values testify that in the case of cyclic voltage modulation *n*_eff_ and/or the thickness-to-charge density ratio differ from the corresponding values which have been found for anodizing at constant voltages. This behaviour is caused by the difference in morphology of ATO structures prepared at constant and periodically changing voltage. To reveal the correlation between morphological parameters of ATO porous structure and anodizing conditions, photonic crystal PC_3 was produced with one thick layer prepared at 60 V in the middle of the periodic layered structure formed at 40 and 60 V. An SEM image of a cleavage of the sample PC_3 is shown in Figure 4a. The cell boundary oxide is weaker than titania nanotubes walls [34]; thus, the fracture propagates mainly through the cell boundaries. Although we cannot see the section of the pores to perform direct measurement of the pore’s diameter, we can draw conclusions about the porosity of the ATO layers formed at various voltages using Z-contrast in SEM images. Assuming that electron density in ATO produced at various voltages is the same, it is clear that the higher brightness in SEM image corresponds to the lower porosity of ATO. Taking into account that the thick layer in the sample PC_3 was formed at 60 V and it appears darker than nearest layers, we can conclude that the porosity of ATO producing at 60 V is higher than porosity of the layers forming at 40 V. Moreover, it becomes evident that other dark layers were formed also at 60 V. It is worth noting that a distance between centers of pores, $D_{\mathrm{int}}$, does not change from the top (Figure S5a) to the bottom (Figure S5b) of the film and pore branching is not observed for ATO obtained in both potentiostatic and cyclic anodizing regimes. In the case of anodizing at constant voltage, $D_{\mathrm{int}}$ increases with voltage ($D_{\mathrm{int}}=$ 82 and 97 nm for 40 and 60 V, respectively, see Figure S6), whereas in the case of periodic voltage modulation interpore distance is close to 84 nm for both types of the layers prepared at 40 and 60 V. Taking into account that interpore distance is constant, the variation of porosity, which is experimentally observed as modulation of Z-contrast in SEM images along the long axis of the pores, is caused by modulation of pores diameter. The same morphology of ATO porous films was observed earlier in [14], whereas a different morphology is common for one-dimensional photonic crystals produced by aluminium anodizing [31,35]. Most likely, the difference in the morphologies of anodic alumina and titania photonic crystals is caused by variation of anodizing regimes. Although anodic alumina porous films can be obtained in both kinetic and diffusion regimes [36], up to now all the anodic alumina photonic crystals were formed in the kinetic regime. In this regime voltage decrease of more than √2 times results in swift pore branching [31,35]. Thus, the effective refractive index of anodic alumina layers formed at a certain voltage during cyclic anodizing is close to *n*_eff_ of anodic alumina layer formed under constant conditions at the same voltage. This behaviour of aluminium anodizing allows one to predict optical properties of photonic crystals on the basis of the properties of samples anodized at constant voltages [31]. In contrast, the diffusion-controlled regime is realised during titanium anodizing in viscous ethylene glycol electrolytes [14,37]. In this case the variation of the voltage of ca. √2 times is not sufficient for porous structure rearrangement (at least at the thickness of transition layer comparable with 1 μm). As a consequence, the morphology and the effective refractive index of the anodic titanium oxide depends on the anodizing regime (constant or modulating voltage). It should be noted that in the case of sufficient thickness of the transition layer, the porous structure of ATO rearranges and pore branching occurs [38].

**Figure 5:**
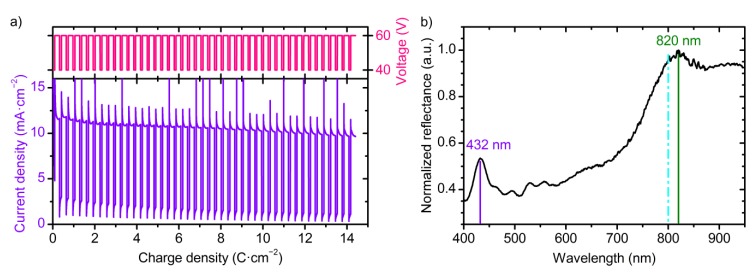
Data for PC_4 with OPL of the layers obtained at 40 and 60 V equal to 0.25*L*_0_ and 0.75*L*_0_, respectively. PC_4 was prepared using Equations (5) and (6) for the OPL calculation. Parameters of the synthesis: *t*_0_ = 70 min; *L*_total_ = 18000 nm; *L*_0_ = 400 nm. (**a**) Dependences of anodizing voltage and current density on charge density. (**b**) Reflectance spectrum at normal incidence of light. Violet and green lines show positions of the first (820 nm) and the second (432 nm) photonic band gaps. Cyan line shows the expected position of the first photonic band gap.

The actual values for the OPL (*n*_eff_(*U, t, λ*)·Δ*d*(Δ*q*, *U*)) of the layers in PC structure prepared at 40 and 60 V are unknown so far. To solve this problem, a sample PC_4 was prepared using Equations (5) and (6) for the OPL calculation and *U*(*L*) profile with assigned OPL of the layers synthesized at 40 and 60 V equal to 0.25*L*_0_ and 0.75*L*_0_, respectively. *U*(*q*) and *j*(*q*) profiles recorded during synthesis of the sample PC_4 are given in Figure 5a. The reflectance spectrum of PC_4 is presented in Figure 5b. Two PBGs can be clearly seen: the first at 820 nm and the second at 432 nm. The difference between the assigned (800 nm) and obtained (820 nm) PBG positions is attributed to the difference between OPL calculated using Equations (5) and (6) and the actual one. The positions of the first and the second PBGs of PC_4 were used to determine the actual dispersion of OPL of the layers prepared at 40 and 60 V in cyclic anodizing regime (see Supplementary Materials “Calculation of OPL” for additional information). Finally, we obtain

(7) $${\mathrm{OPL}}^{m}\left(\Delta q,\lambda ,U=40V\right)\;=\;\Delta q\cdot \left(A_{40}^{m}+\;\frac{B_{40}^{m}}{\lambda^{2}}\right),$$

(8) $${\mathrm{OPL}}^{m}\left(\Delta q,\lambda ,U=60V\right)\;=\;\Delta q\cdot \left(A_{60}^{m}+\;\frac{B_{60}^{m}}{\lambda^{2}}\right),$$

where $A_{40}^{m}$ = 1057 nm·cm^2^·C^−1^; $B_{40}^{m}$ = 3.040 × 10^7^ nm^3^·cm^2^·C^−1^; $A_{60}^{m}$ = 1358 nm·cm^2^·C^−1^; and $B_{60}^{m}$ = 1.305 × 10^7^ nm^3^·cm^2^·C^−1^. Here and below, parameters for the case of modulating voltage are marked by superscript index m.

**Figure 6:**
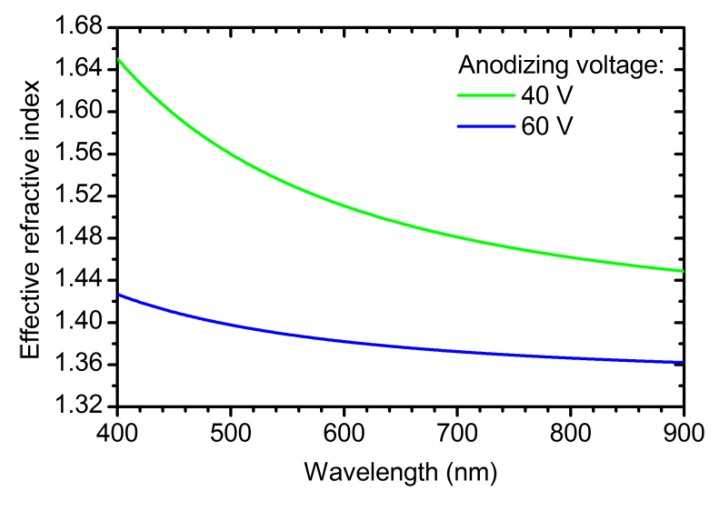
Cauchy dispersion (*n*^m^(*λ*) = *A*^m^ + *B*^m^/*λ*^2^) of the effective refractive index of the photonic crystal layers obtained at different anodizing voltage: 40 V (green line, *A*^m^ = 1.399, *B*^m^ = 40,260 nm^2^) and 60 V (blue line, *A*^m^ = 1.346, *B*^m^ = 12,930 nm^2^).

**Figure 7:**
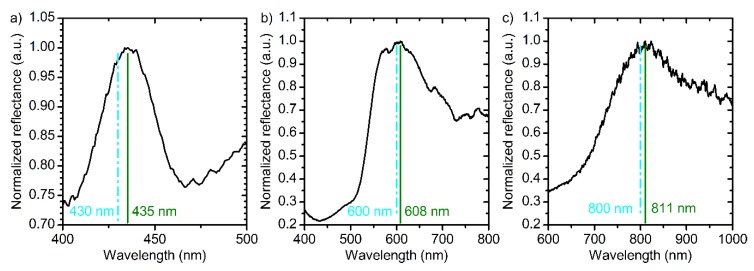
Reflectance spectra at normal incidence of light of PC_5 (**a**), PC_6 (**b**), and PC_7 (**c**) prepared by the suggested technique using Equations (7) and (8) for the OPL calculation. The assigned positions of photonic band gap are 430 (**a**), 600 (**b**), and 800 nm (**c**). Cyan dash lines and green solid lines show the assigned and the measured photonic band gap positions. Parameters of the synthesis: (**a**) *t*_0_ = 50 min; *L*_total_ = 10,750 nm; *L*_0_ = 215 nm; (**b**) *t*_0_ = 60 min; *L*_total_ = 15,000 nm; *L*_0_ = 300 nm; (**c**) *t*_0_ = 70 min; *L*_total_ = 16,000 nm; *L*_0_ = 400 nm.

Although the information about the OPL is sufficient for controlling PBG position, *n*_eff_ and Δ*d* are of great interest. To obtain them, brightness profiles of the SEM images were used (Figure 4b). These profiles allow one to measure the layers thickness and, consequently, to determine Δ*d*. Then, dispersion of *n*_eff_ for the PCs layers prepared under different voltages (Figure 6) was calculated by dividing the corresponding value of the OPL (Equations (7) and (8)) by the measured thickness (see Supplementary Materials “Calculation of *n*_eff_” for additional information):

(9) $$\Delta d^{m}\left(\Delta q,\;U\right)=\Delta q\cdot \left(d_{q}^{m}+d_{\mathrm{qU}}^{m}\cdot U\right),$$

where $d_{q}^{m}$ = 247 nm·cm^2^·C^−1^, $d_{\mathrm{qU}}^{m}$ = 12.7 nm·cm^2^·C^−1^·V^−1^.

(10) $$n_{\mathrm{eff}}^{m}\left(U,t_{e},\lambda \right)=n_{0}^{m}-n_{U}^{m}U+\;\frac{B_{\lambda }-B_{U\lambda }U\;}{\lambda^{2}}-n_{t}^{c}t_{e}^{2}+n_{\mathrm{Ut}}^{c}Ut_{e}^{2},$$

where $n_{0}^{m}$ = 1.505; $n_{U}^{m}$ = 2.65 × 10^−3^ V^−1^; *B*_λ_ = 94920 nm^2^; and *B*_Uλ_ = 1367 nm^2^V^−1^. It was assumed that the rate of decrease in the effective refractive index of anodic titanium oxide in electrolyte solution is the same for anodizing at constant and modulating voltage (for the values of $n_{t}^{c}$ and $n_{\mathrm{Ut}}^{c}$ please refer to Equation (4)). To confirm that Equations (9) and (10) describe *n*_eff_ and thickness of the PCs layers precisely, samples PC_5, PC_6, and PC_7 were prepared with different assigned positions of PBG: 430, 600, and 800 nm, respectively. The reflectance spectra of these PCs are presented in Figure 7. It is apparent that for all samples the assigned PBG position is in a good agreement with the acquired one with the accuracy better than 98.5%. Therefore, our method allows one to control PBG position at least in the interval from 430 nm to 800 nm. The applicability of the suggested approach in the region below 430 nm is limited due to the absorption of light by titanium oxide in ultraviolet spectral range [39]. On the other hand, there are no restrictions to use the suggested method to control PBG position above 800 nm. To the best of our knowledge, the precise control of the PBG position of ATO PCs has never been reported before.

**Figure 8:**
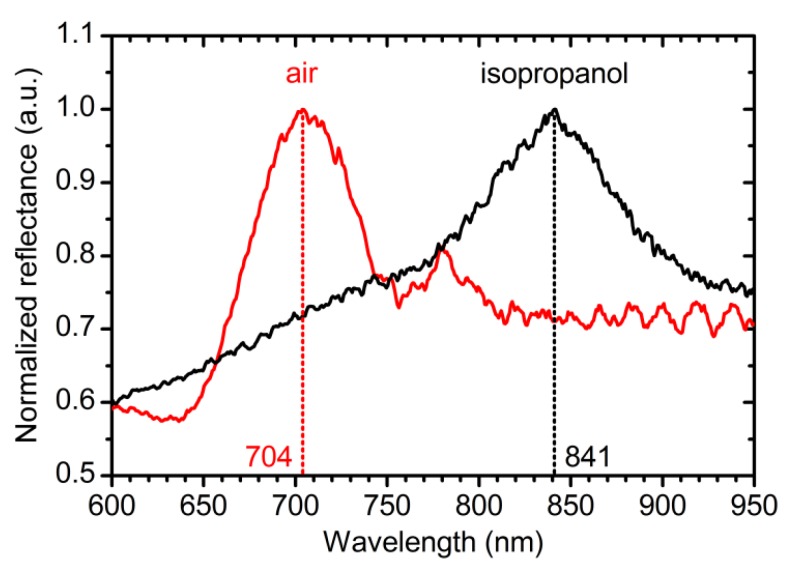
Refractive index sensor ability of the anodic titanium oxide (ATO) photonic crystals. Reflectance spectra of PC_8 with pores filled with air (red line) and isopropanol (black line). Parameters of the synthesis: *t*_0_ = 150 min; *L*_total_ = 21,000 nm; *L*_0_ = 350 nm.

The obtained PCs were tested as refractive index sensors. It can be seen in Figure 8 that the position of PBG is sensitive to the refractive index of the pore filling substance in accordance with effective medium theory [19]. Red shift of the PBG position by 137 nm is observed after pore filling with isopropanol (*n*_isopropanol_ = 1.38). The sensitivity of such a sensor is 360 nm/RIU, where RIU is a refractive index unit. It is worth noting, that the achieved value of the sensitivity is much higher than the corresponding value for ATO refractive index sensors (about 250 nm/RIU) reported earlier [11,15].

## 4. Conclusions

Anodizing with voltage versus optical path length modulation was realized for the fabrication of anodic titanium oxide photonic crystals with precise PBG position for the first time. The developed technique allows one to control the position of the photonic band gap of anodic titanium oxide photonic crystal in the range of 430–800 nm with an accuracy better than 98.5%. The effective refractive index of anodic titanium oxide films prepared at constant voltage of 40 or 60 V (1.66–1.77 at 800 nm) is higher than *n*_eff_ of ATO PCs formed under periodic square-wave modulation of anodizing voltage between 40 and 60 V (1.37–1.46 at 800 nm). In both cases, ATO layers obtained at 40 V have higher *n*_eff_ than at 60 V. ATO photonic crystals are promising as the refractive index sensors owing to the high sensitivity of the photonic band gap position to the refractive index of a liquid filling the pores.

## Supplementary Materials

The following are available online at https://www.mdpi.com/2079-4991/9/4/651/s1, Figure S1: Scheme of electrochemical cell for titanium anodizing, Figure S2: Reflectance spectra of ATO porous films obtained at 40 V, Figure S3: Reflectance spectra of ATO porous films obtained at 60 V, Figure S4: SEM images of cleavages of the ATO porous films prepared at constant voltage of 40 V and 60 V, Figure S5: SEM images of the top and the bottom surfaces of the sample PC_3, Figure S6: SEM images of the bottom surfaces of the samples prepared at constant voltage of 40 V and 60 V.
