# Supplementary material for: Titania Photonic Crystals with Precise Photonic Band Gap Position via Anodizing with Voltage versus Optical Path Length Modulation
Source: Nanomaterials (Basel). 2019 Apr 23;9(4):651. doi: 10.3390/nano9040651 (PMC6523195; doi:10.3390/nano9040651)
Supplement: Supplementary file 1 [file nanomaterials-09-00651-s001.pdf]

Supplementary Materials: Titania Photonic crystals with Precise Photonic Band Gap Position via Anodizing with Voltage versus Optical Path Length Modulation

Georgy A. Ermolaev, Sergey E. Kushnir *, Nina A. Sapoletova and Kirill S. Napolskii

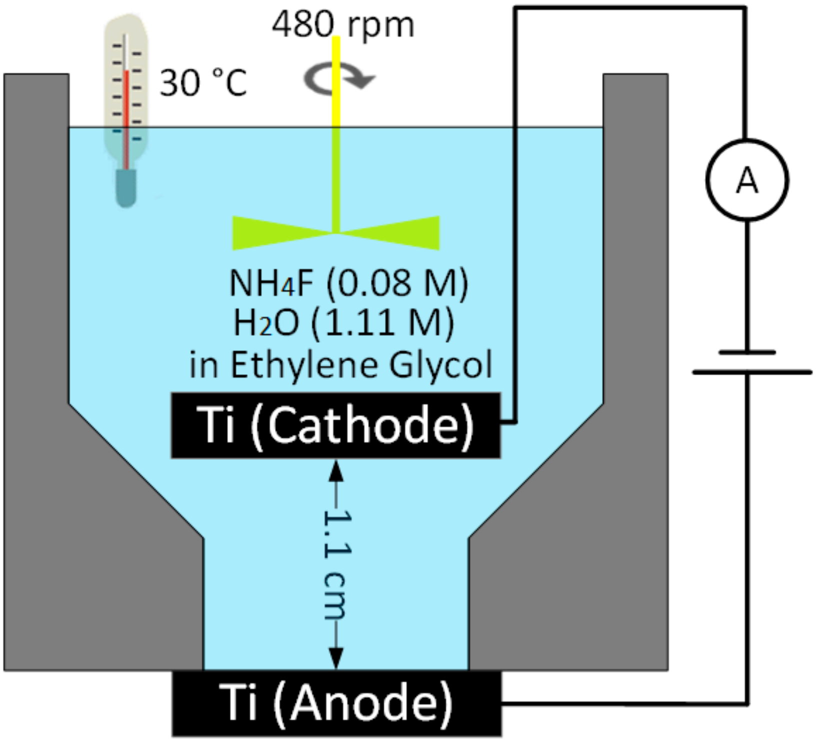

Figure S1. Scheme of electrochemical cell for titanium anodizing.

Reflectance Spectra

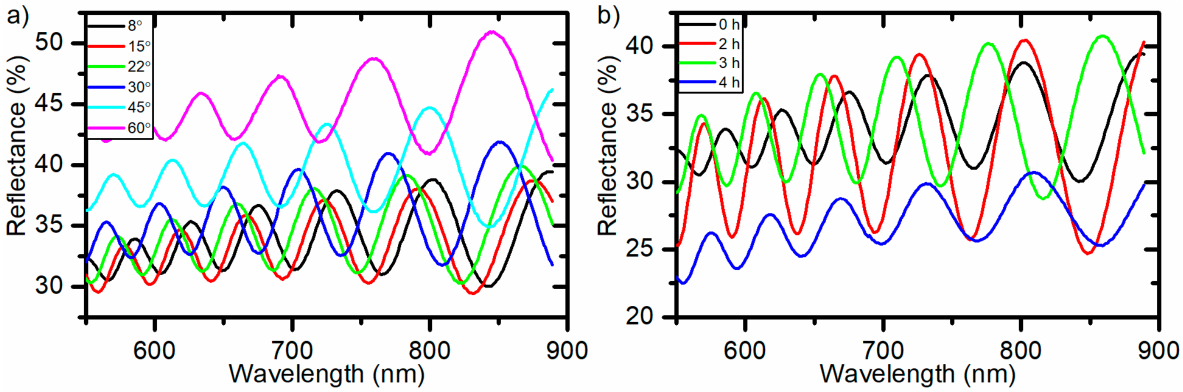

Figure S2. Reflectance spectra of ATO porous films obtained at 40 V. (**a**) Spectra of the sample without additional etching recorded at different angles of incidence. (**b**) Spectra of the samples etched in electrolyte solution for various time durations. Data is collected at the angle of incidence of 8°.

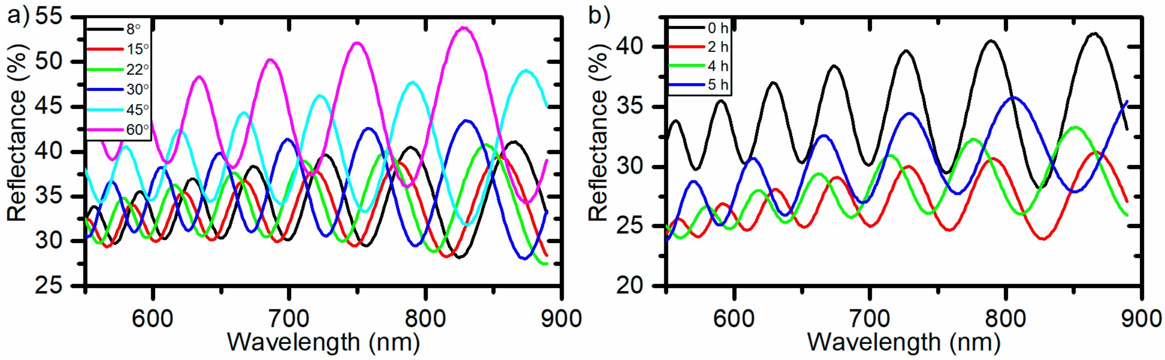

Figure S3. Reflectance spectra of ATO porous films obtained at 60 V. (**a**) Spectra of the sample without additional etching recorded at different angles of incidence. (**b**) Spectra of the samples etched in electrolyte solution for various time durations. Data is collected at the angle of incidence of 8°.

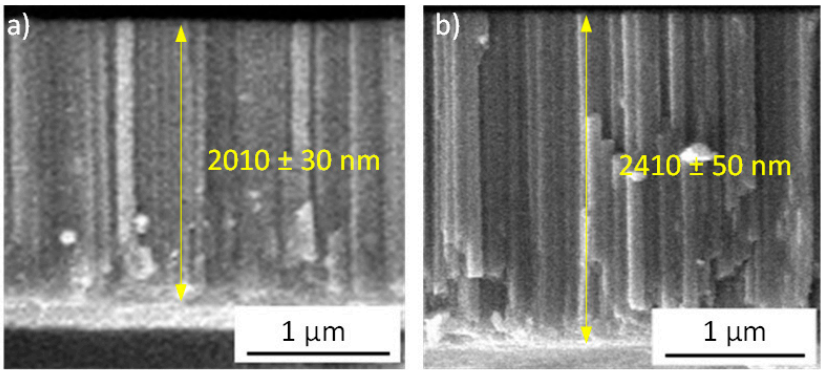

Figure S4. SEM images of cleavages of the ATO porous films prepared at constant voltage of 40 V (**a**) and 60 V (**b**). Anodizing was stopped when the charge density reached 2.33 C cm^{-2}. Data for the samples after additional etching in electrolyte during 2 hours is presented. According to reflectance spectra, the thicknesses of the ATO porous films presented in panels (**a**) and (**b**) are 2040 ± 60 nm and 2460 ± 170 nm, respectively.

On ATO Porosity

In the assumption that the refractive index of the pore walls of the ATO films is independent of voltage, the difference between the effective refractive indices of the films obtained at various voltages is caused solely by variation of porosity. Assuming 2D hexagonally packed cylindrical pores [1] and linear relation ($D_p(U) = kU$) of pore diameter D_p with respect to voltage U we obtain:

$$p = \frac{\pi}{2\sqrt{3}}\left[\frac{D_p(U)}{D_{int}(U)}\right]^2 = \frac{\pi}{2\sqrt{3}}\left[\frac{kU}{D_{int}(U)}\right]^2 \quad \text{(S1)}$$

where $D_{int}(U)$ is interpore distance of ATO film formed at voltage U.

According to SEM images of the bottom of the ATO films, $D_{int}(U)$ is equal to 82 ± 1 and 97 ± 1 nm for the samples formed at 40 and 60 V, respectively. Therefore, from Equation (S1) we obtain the ratio of porosities for the films prepared at 40 and 60 V:

$$\frac{p_{60}}{p_{40}} = \left[\frac{D_{int}(40)}{D_{int}(60)}\right]^2\left[\frac{60}{40}\right]^2 \approx 1.6 \quad \text{(S2)}$$

Hence, porosity for the layer obtained at 60 V is larger than for the one prepared at 40 V, and, as a result, the effective refractive index is smaller in accordance with the effective medium theory.

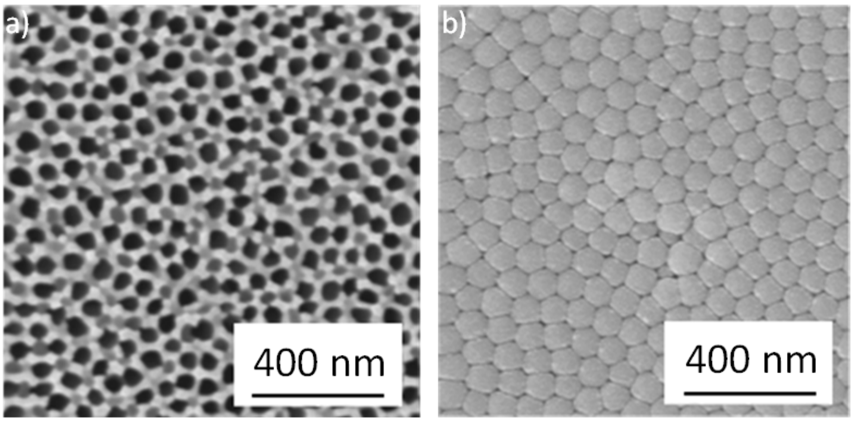

Figure S5. SEM images of the top (**a**) and the bottom (**b**) surfaces of the sample PC_3.

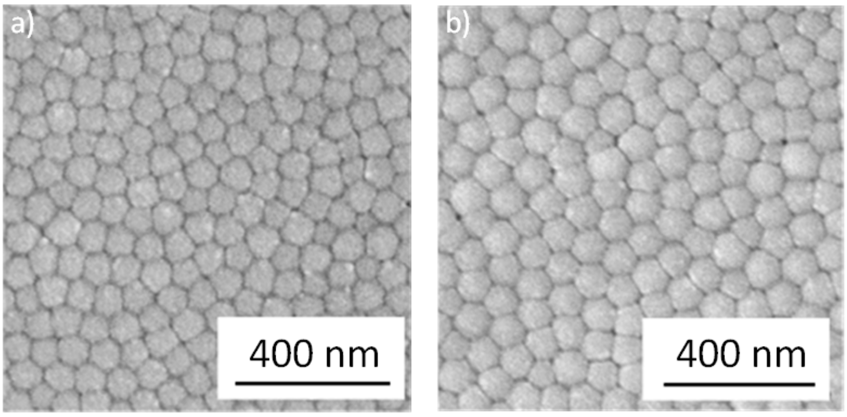

Figure S6. SEM images of the bottom surfaces of the samples prepared at constant voltage of 40 V (**a**) and 60 V (**b**). The samples were stored in electrolyte during 2 hours after the end of anodizing.

Calculation of OPL

PC_4 has two photonic band gaps at λ_1 = 820 nm and λ_2 = 432 nm. As Equations (5) and (6) gives correct calculation of the L_0 of the ATO photonic crystal in the case of equal OPL for the layers prepared at 40 and 60 V, then the deviation of the actual OPL from the calculated one should be the opposite values (x and $-x$). These deviations could be calculated using Equations (1), (5) and (6) in the following way:

$$\lambda_1 = 2 \cdot \left(3p_{60}^{\mathrm{m}}(\lambda_1) + p_{40}^{\mathrm{m}}(\lambda_1)\right) = 2 \cdot \left(3\left(p_{60}^{*}(\lambda_1) + x(\lambda_1)\right) + \left(p_{40}^{*}(\lambda_1) - x(\lambda_1)\right)\right) = 2 \cdot \left(3p_{60}^{*}(\lambda_1) + p_{40}^{*}(\lambda_1)\right) + 4x(\lambda_1) = 800 + 4x(\lambda_1) \rightarrow x(\lambda_1) = \frac{\lambda_1 - 800}{4} = 5 \text{ nm}$$

$$2\lambda_2 = 2 \cdot \left(3p_{60}^{\mathrm{m}}(\lambda_2) + p_{40}^{\mathrm{m}}(\lambda_2)\right) = 2 \cdot \left(3\left(p_{60}^{*}(\lambda_2) + x(\lambda_2)\right) + \left(p_{40}^{*}(\lambda_2) - x(\lambda_2)\right)\right) \rightarrow x(\lambda_2) = \frac{\lambda_2 - 3p_{60}^{*}(\lambda_2) - p_{40}^{*}(\lambda_2)}{2} = \frac{\lambda_2 - 430}{2} = 1 \text{ nm}$$

where $3p_{60}^{\mathrm{m}}(\lambda)$ is optical path length (for wavelength λ) of the layer obtained at 60 V; $p_{40}^{\mathrm{m}}(\lambda)$ is optical path length (for wavelength λ) of the layer obtained at 40 V; $3p^{*}(\lambda)$ is optical path length (for wavelength λ) obtained at 60 V, according to Equation (6); $p^{*}(\lambda)$ is optical path length (for wavelength λ) obtained at 40 V, according to Equation (5).

As a result, the actual optical path length of the layers prepared at 40 and 60 V under modulated voltage are:

$$\mathrm{OPL^m}(\Delta q, \lambda = 820\ \mathrm{nm}, U = 40\ \mathrm{V}) = \Delta q \cdot \left(A^*_{40} + \frac{B^*_{40}}{820^2}\right)\frac{p^*(\lambda_1) - x(\lambda_1)}{p^*(\lambda_1)} = \Delta q \cdot 1102\ \mathrm{nm{\cdot}cm^2{\cdot}C^{-1}}$$

$$\mathrm{OPL^m}(\Delta q, \lambda = 432\ \mathrm{nm}, U = 40\ \mathrm{V}) = \Delta q \cdot \left(A^*_{40} + \frac{B^*_{40}}{432^2}\right)\frac{p^*(\lambda_2) - x(\lambda_2)}{p^*(\lambda_2)} = \Delta q \cdot 1219\ \mathrm{nm{\cdot}cm^2{\cdot}C^{-1}}$$

$$\mathrm{OPL^m}(\Delta q, \lambda = 820\ \mathrm{nm}, U = 60\ \mathrm{V}) = \Delta q \cdot \left(A^*_{60} + \frac{B^*_{60}}{820^2}\right)\frac{p^*(\lambda_1) + x(\lambda_1)}{p^*(\lambda_1)} = \Delta q \cdot 1377\ \mathrm{nm{\cdot}cm^2{\cdot}C^{-1}}$$

$$\mathrm{OPL^m}(\Delta q, \lambda = 432\ \mathrm{nm}, U = 60\ \mathrm{V}) = \Delta q \cdot \left(A^*_{60} + \frac{B^*_{60}}{432^2}\right)\frac{p^*(\lambda_2) + x(\lambda_2)}{p^*(\lambda_2)} = \Delta q \cdot 1428\ \mathrm{nm{\cdot}cm^2{\cdot}C^{-1}}$$

Taking account of Cauchy form dispersion of the effective refractive index, we finally have parameters given in Equations (7) and (8).

Calculation of n_{eff}

Equation (9) was obtained using the experimental values of the thickness of the ATO layers (Figure 4) and charge density values obtained from the integration of the current density over time, assuming the linear dependence on applied voltage. The thicknesses of the layers formed at 40 V are 343 nm, whereas the thickness of central ATO layer formed at 60 V is 1068 nm. The charge density increment is 0.454 C·cm^{-2} for the layer formed at 40 V and 1.058 C·cm^{-2} for the layer obtained at 60 V.

Equation (10) was obtained by dividing Equations (7) and (8) by Equation (9), assuming the linear dependence of the effective refractive index on the applied voltage:

$$n^{\mathrm{m}}_{\mathrm{eff}}(U = 40\ V, t_{\mathrm{e}} = 0\ h, \lambda) = \frac{\Delta q \cdot \left(A^{\mathrm{m}}_{40} + \frac{B^{\mathrm{m}}_{40}}{\lambda^2}\right)}{\Delta q \cdot \left(d^{\mathrm{m}}_{\mathrm{q}} + d^{\mathrm{m}}_{\mathrm{qU}} \cdot U\right)} = \frac{1057 + \frac{3.04 \cdot 10^7}{\lambda^2}}{247 + 12.7 \cdot 40} = 1.399 + \frac{40260}{\lambda^2}$$

$$n^{\mathrm{m}}_{\mathrm{eff}}(U = 60\ V, t_{\mathrm{e}} = 0\ h, \lambda) = \frac{\Delta q \cdot \left(A^{\mathrm{m}}_{60} + \frac{B^{\mathrm{m}}_{60}}{\lambda^2}\right)}{\Delta q \cdot \left(d^{\mathrm{m}}_{\mathrm{q}} + d^{\mathrm{m}}_{\mathrm{qU}} \cdot U\right)} = \frac{1358 + \frac{1.305 \cdot 10^7}{\lambda^2}}{247 + 12.7 \cdot 40} = 1.346 + \frac{12930}{\lambda^2}$$

References

1. Macak, J.M.; Tsuchiya, H.; Ghicov, A.; Yasuda, K.; Hahn, R.; Bauer, S.; Schmuki, P. TiO_2 nanotubes: Self-organized electrochemical formation, properties and applications. *Curr. Opin. Solid State Mater. Sci.* **2007**, *11*, 3–18; DOI:10.1016/j.cossms.2007.08.004.
